# Do you Play or Do you Train? Insights From Individual Sports for Training Load and Injury Risk Management in Team Sports Based on Individualization

**DOI:** 10.3389/fphys.2020.00995

**Published:** 2020-08-21

**Authors:** Daniel Boullosa, Arturo Casado, João Gustavo Claudino, Pedro Jiménez-Reyes, Guillaume Ravé, Adrián Castaño-Zambudio, Adriano Lima-Alves, Silvio Assis de Oliveira, Gregory Dupont, Urs Granacher, Hassane Zouhal

**Affiliations:** ^1^Graduate Program of Movement Sciences, INISA, Federal University of Mato Grosso do Sul, Campo Grande, Brazil; ^2^Faculty of Health Sciences, Isabel I University, Burgos, Spain; ^3^School of Physical Education and Sport, São Paulo University, São Paulo, Brazil; ^4^Center for Sport Studies, Rey Juan Carlos University, Madrid, Spain; ^5^Toulouse Football Club, Toulouse, France; ^6^Department of Sport Sciences, University Federal of Minas Gerais, Belo Horizonte, Brazil; ^7^Real Madrid C.F, Madrid, Spain; ^8^Division of Training and Movement Sciences, University of Potsdam, Postdam, Germany; ^9^Department of Sport Sciences, University of Rennes, Rennes, France

**Keywords:** training monitoring, injury prevention, physical fitness, acute:chronic workload ratio, periodization, sport physiology, evolutionary biology

## Introduction

The understanding of the potential causes of musculoskeletal injuries in any competitive sport needs to address their multifactorial nature, which results from complex associations among different external conditions and modifiable and non-modifiable intrinsic risk factors (Drew and Purdam, 2016; Kalkhoven et al., 2020a). In this context, the cause of any non-contact injury merely results from a sum of loads generating a force that exceeds the limit supported by the respective biological tissue (Zernicke and Whiting, 2008). Consequently, it has been suggested that a poor load management is a major risk factor for injury in sport settings (Gabbett, 2016).

One novel monitoring tool for injury risk management is the acute: chronic workload ratio (ACWR). The ACWR is currently in the spot light of sport sciences (Griffin et al., 2020; Maupin et al., 2020). While some emerging evidence suggests that it is a valid method to identify an increased injury risk (Andrade et al., 2020), other authors have pointed out its methodological limitations and even questioned its validity (Impellizzeri et al., 2020; Wang et al., 2020). Proponents of the ACWR approach argue that athletes are at greater risk of sustaining a time-loss injury when the ACWR is higher relative to a lower or moderate ACWR (Andrade et al., 2020). In other words, the ACWR helps to identify critical windows in terms of elevated injury risk based on imbalanced training loading as for example sudden spike loads (Bowen et al., 2020).

The ACWR supposedly follows the classical fitness-fatigue model (Banister et al., 1975). Paradoxically, the rationale for the ACWR resides on several assumptions that are not in agreement with expected coaching practices as, for instance, progressive loading (Foster et al., 1995). Further, the occurrence of a sudden spike, in the context of any sport, could be simply interpreted as a training load error (Drew and Purdam, 2016; Kalkhoven et al., 2020b). In this respect, it is important to note that the ACWR literature includes mostly team sport studies, with the exception of a few studies in individual sports (Collette et al., 2018; Myers et al., 2020). For instance, a single study in swimming found the Acute Recovery and Stress Scale (ARSS) to be more valid than the ACWR to monitor recovery-stress (Collette et al., 2018). In another recent study in junior tennis it was suggested that ACWR and injury history were the best predictors of injury incidence (Myers et al., 2020). It should be noted that tennis is an individual sport with the players directly interacting with each other, therefore it shares some mutual characteristics with most team sports. These mutual characteristics may include a number of contextual factors such as score line, match location, opposition standard, moment of the season, playing formation, player's role (Paul et al., 2015; Rago et al., 2019a; Curtis et al., 2020), and playing surface (Rago et al., 2019b; Vescovi and Falenchuk, 2019). For this reason, in this article we will refer to individual sports as those sports in which single athletes do not experience direct opposition from their opponents during competitions, with performances being strongly linked to physiological capacity (e.g., track and field or swimming). In this context, the limited number of ACWR studies in individual sports may be therefore not casual thus suggesting an inappropriate load management in some team sports under certain circumstances, probably related to the complexity associated to the existence of contextual factors which, in turn, would influence the physical and physiological profiles of team sport athletes.

Therefore, the aim of this opinion article is to provide a rationale based on coaching practices and scientific evidence from individual and team sports, that may help to better understand and, subsequently improve, training loads and injury risk management in team sports.

## Differences in Physiological Demands Between Individual and Team Sports

The first consideration to be highlighted are differences in physiological demands between individual and team sports. While individual and team sports share some physiological attributes in common (Degens et al., 2019), it is noteworthy that individual sports need to maximize specific capacities (e.g., maximum oxygen consumption [VO_2_max], anaerobic threshold and economy in endurance sports) to be successful in competition. In team sports, athletes do not need to develop their maximal but optimal physiological attributes (e.g., VO_2_max, muscle power) due to the multifactorial nature of performance determinants (e.g., technical skills, physical qualities, tactical behavior) that are ultimately decisive for success (Boullosa and Abreu, 2014). This differentiation is in agreement with the biological principle of allocation (Van Damme et al., 2002; Boullosa et al., 2013b) which, in turn, explains the important differences among sports that are evident when examining normative values of several physiological parameters (e.g., VO_2_max in endurance athletes vs. soccer players) (Tønnessen et al., 2013; Sandbakk and Holmberg, 2017). Indeed, the need of maximizing specific physiological attributes in individual sports reveals a direct link between physiological characteristics and performance. This may explain why performance prediction works better with individual compared with team sports using data from physiological testing (Padilla et al., 2000; Esteve-Lanao et al., 2019).

Another important difference between individual vs. team sports refers to the competitive calendars which are often denser in team sports, with frequent competitions throughout the year (Nassis et al., 2020). In contrast, in individual sports, the competitive periods are generally shorter and allocated to specific periods (e.g., summertime) of the season (Costa et al., 2010). This important difference modulates two key loading events, namely peaking and tapering, which are directly linked to periodization models (Timpka et al., 2020). Thus, in individual sports, there are more prolonged periods (i.e., macrocycles) for loading before peaking, which usually occurs a few times over the year after important workload accumulation (Tønnessen et al., 2013). In team sports, a time-restricted workload accumulation normally occurs during the pre-season (Boullosa et al., 2013a) of the regular season, or during camps before tournaments (Noor et al., 2019). Therefore, there is no “true peaking” at any time of the season in team sports. Instead, there is a performance plateau on the level of physical and physiological adaptations that allows appropriate technical-tactical performances over time. These different loading patterns are also mirrored in tapering strategies. In individual sports, coaches frequently use tapering to reduce the workload before competition (Bosquet et al., 2007). In team sports, tapering is less pronounced and limited to some specific events during the season such as at the end of the pre-season or before an important match (Vachon et al., 2020). In this context, it seems important to postulate that the reduced loads on the days before matches in team sports cannot be considered as tapering. This weekly practices mostly reflect an attempt to recover sufficiently from matches, which is more evident in periods of congested match play (Saidi et al., 2019).

Another key point refers to the current understanding of the complex and self-correcting nature of the periodization process. That is, contrary to the static and fixed application of traditional periodization models, modern periodization approaches (Kiely, 2012, 2018) are more based on the adaptation of training loads to the readiness and the levels of fatigue and fitness exhibited by the individual athlete. This type of periodization strategy affords different sources of data from the individual (e.g., heart rate, session rating of perceived exertion) and is adapted on a daily basis following biological principles (Kiely, 2012; Boullosa and Nakamura, 2013; Boullosa et al., 2013b). This is important to consider given that this level of individualization is mandatory in individual sports for success. In team sports, it is less common because training workloads are most often collectively performed, with predefined daily objectives included in the weekly microcycle. Furthermore, while traditional periodization approaches only consider the management of training and competitive workloads, a multifactorial approach has recently been suggested which includes more periodization components as recovery strategies, psychological skills, nutrition and skill acquisition (Mujika et al., 2018). All these periodization components are simultaneously manipulated to meet the desired short- and long-term adaptations of athletes.

The relationship between internal and external training loads (Impellizzeri et al., 2019) is a relevant difference between both types of sport (team vs. individual) which directly affects the quality of training monitoring. Thus, the relationship between internal and external loads is easier to monitor in individual sports because of their direct relationship (e.g., power or velocity vs. heart rate) (Boullosa et al., 2020). In team sports, it is more complicated because of the influence of contextual factors (Brito et al., 2016; Oliva-Lozano et al., 2020) affecting both internal and external load parameters in a different manner (Fox et al., 2020). The picture is even more complex if we consider that the current ACWR literature has found different relationships between ACWR indices generated with different internal [e.g., session rating of perceived exertion (sRPE)] and external (e.g., accelerations) load parameters in team sports characterized by different psycho-physiological demands (Andrade et al., 2020; Griffin et al., 2020). This means that the relationships found between ACWR and injury risk are not interchangeable among team sports, therefore suggesting the greater weight of extrinsic factors within this relationship, as it has been also suggested that the strength of the relationships between internal and external load parameters in team sports depends upon the training mode (McLaren et al., 2018).

## The Individualization Approach in Team Sports

After having presented the main differences between individual and team sports, we suggest an individualized approach to better manage training loads in team sports which may help to reduce the likelihood of sustaining injuries and optimize performance. Thus, individualization is the key in a multifactorial periodization model. In other words, the individual response in terms of training and competitive workload, nutrition, psychological skills, recovery strategies, and skill acquisition, allows a more flexible periodization approach on a daily basis. Moreover, given that injury history of an individual is a relevant factor for the relationship between workloads and injury risk (Esmaeili et al., 2018), medical staff together with strength and conditioning coaches should collectively develop individualized preventive and therapeutic interventions for those players who suffered injuries in the past, with special attention to the context (Bolling et al., 2018).

Although a significant time of daily training in team sports is devoted to collective training, individualization of all these aspects would result in a better control of the fitness-fatigue relationship by avoiding any sudden workload spike and thus an increased injury risk. Further, as technological advances allow to monitor the individual player during collective training (e.g., measuring the number of accelerations during small sided games with GPS technologies), individualization of all the components of a multifactorial periodization during both individual and collective behaviors appears feasible (Li et al., 2016). However, the excess of information to be managed with this approach is extremely high, therefore the use of big data analysis through artificial intelligence is warranted (Claudino et al., 2019). This is especially true in big clubs, in which the different staff divisions need to better coordinate their work (e.g., medical staff, strength and conditioning specialists, physiotherapists, analysts, nutritionist, etc.) through fluent communication (Bolling et al., 2020) to successfully manage the individualization process. When gathering all these “objective” data, head coaches' expertise, and knowledge should not be ignored for decision making. However, head coaches must be prepared and well-supported by different staff divisions as the amount of data to be managed is continuously increasing, including the monitoring of daily living activities (Boullosa et al., 2013b; Düking et al., 2018; Izzicupo et al., 2019).

**Figure 1:**
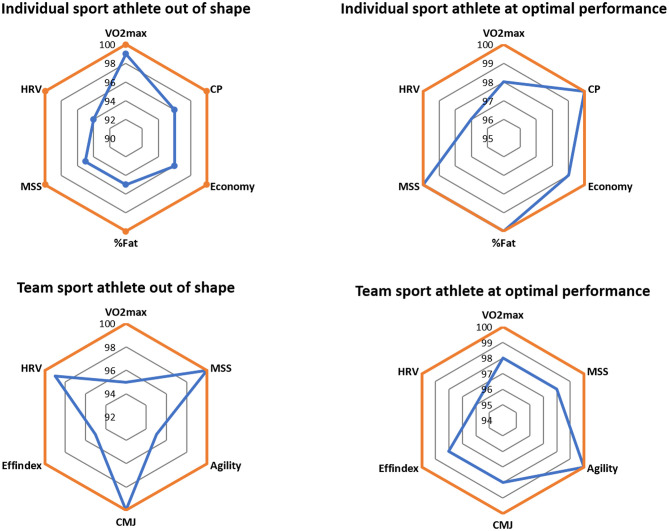
Simulated example of the different evolution of physiological profiles in individual vs. team sports athletes. Note that in individual sports, most physiological parameters tend to be at their maximum individual values (personal best) at optimal performances. In contrast, this is not necessary for optimal performances in team sports. CMJ, countermovement jump; CP, critical power; Effindex, efficiency index; HRV, heart rate variability; MSS, maximum sprinting speed; VO_2_max, maximum oxygen consumption; %Fat, percentage of body fat.

The major advantage of this individualized approach is that it avoids any excessive loading, and therefore sudden individual workload spikes. Accordingly, the ACWR could be simply used to confirm an inappropriate individual workload management which would never occur following this approach. This aspect is one key to better understand the questioned validity of the ACWR (Impellizzeri et al., 2020). Using this individualized monitoring approach, it is possible to prevent excessive fatigue, incomplete recovery or insufficient readiness associated with low fitness. Similarly, the potential uncoupling between selected internal and external workload parameters due to the influence of contextual factors during matches (Fox et al., 2020), can be better managed with the consideration of sport specific contextual factors (e.g., starters vs. non-starters) to better adapt selected periodization components (e.g., psychological skills, recovery strategies) and thus, to avoid any individual load imbalance. Meanwhile, it is still to be resolved if the identification of individual physiological profiles associated with optimal technical and tactical performances, would assist to better select individualized interventions to mimic these optimal individual profiles throughout the season. This is contrary to the direct link between maximal physiological test performances and competitive performance outcomes in most individual sports. For instance, in team sports, maximal leg power is not necessarily linked with match performance (Tangalos et al., 2015). This is different with individual sports such as sprinting (Loturco et al., 2015). An example of this different evolution between physiological profiles in individual vs. team sports is shown in Figure 1.

There are a number of monitoring tools and parameters to assist in this individualization process such as aerobic power (Milanez et al., 2011), the end speed of the 30-15 intermittent fitness tests (Malone et al., 2018a; Grgic et al., 2020), the anaerobic speed reserve (Buchheit and Mendez-Villanueva, 2014), muscle power (Loturco et al., 2019), heart rate variability (Rave et al., 2018, 2019), countermovement jump performance (Claudino et al., 2012), the force-velocity profile (Mendiguchia et al., 2016), HR measures during specific drills (Lacome et al., 2018), well-being questionnaires (Malone et al., 2018b), or individualized training loads modeling (Bartlett et al., 2017) among others. Specific monitoring tools should be selected and adapted to each sport and setting while developing the athlete monitoring cycle (Gabbett et al., 2017). However, this process is incomplete if the periodization components are not simultaneously adapted to meet the desired individual adaptation of each athlete. Meanwhile, it needs to be solved if the individualization of some monitoring tools is really necessary (Scott and Lovell, 2018), or if it is simply a problem of signal-to-noise ratio (Boullosa and Abreu, 2014).

In summary, we have illustrated the main differences regarding workload management between individual and team sports, and how a true individualization of all the periodization factors in team sports could result in a better managed fitness-fatigue equilibrium and, thus, in a reduced injury risk and enhanced performance. Future studies should illustrate how this paradigm may induce better performances and health outcomes in team sport athletes.

## Author Contributions

DB conceived and designed the idea. All authors wrote, revised the manuscript draft, read, and approved the final manuscript version.

## Conflict of Interest

The authors declare that the research was conducted in the absence of any commercial or financial relationships that could be construed as a potential conflict of interest.
